# Hospitalisations with infections related to antimicrobial-resistant bacteria from the French nationwide hospital discharge database, 2016

**DOI:** 10.1017/S0950268819000402

**Published:** 2019-03-12

**Authors:** M. Opatowski, P. Tuppin, K. Cosker, M. Touat, G. De Lagasnerie, D. Guillemot, J. Salomon, C. Brun-Buisson, L. Watier

**Affiliations:** 1 Biostatistics, Biomathematics, Pharmacoepidemiology and Infectious Diseases (B2PHI), Inserm, UVSQ, Institut Pasteur, Université Paris-Saclay, Paris, France; 2 Caisse nationale de l'assurance maladie, Direction de la stratégie des études et des statistiques, Paris, France; 3 Département Biostatistiques, Santé Publique, Information médicale, AP-HP Hôpitaux Universitaires Pitié Salpêtrière – Charles Foix, Paris, France; 4 Laboratoire Interdisciplinaire d’évaluation des Politiques Publiques, Paris, France; 5 AP-HP, Hôpital Raymond-Poincaré, Garches, France

## Abstract

Massive use of antibiotics has led to increased bacterial resistance to these drugs, making infections more difficult to treat. Few studies have assessed the overall antimicrobial resistance (AMR) burden, and there is a paucity of comprehensive data to inform health policies. This study aims to assess the overall annual incident number of hospitalised patients with AMR infection in France, using the National Hospital Discharge database. All incident hospitalisations with acute infections in 2016 were extracted. Infections which could be linked with an infecting microorganism were first analysed. Then, an extrapolation of bacterial species and resistance status was performed, according to age class, gender and infection site to estimate the total number of AMR cases. Resistant bacteria caused 139 105 (95% CI 127 920–150 289) infections, resulting in a 12.3% (95% CI 11.3–13.2) resistance rate. ESBL-producing Enterobacteriaceae and methicillin-resistant *Staphylococcus aureus* were the most common resistant bacteria (>50%), causing respectively 49 692 (95% CI 47 223–52 142) and 19 493 (95% CI 15 237–23 747) infections. Although assumptions are needed to provide national estimates, information from PMSI is comprehensive, covering all acute bacterial infections and a wide variety of microorganisms.

## Introduction

Antibiotics have markedly reduced the mortality associated with infectious diseases in the 20th century. However, their remarkable efficacy has been accompanied by extensive use in human and animal medicine: worldwide antibiotic consumption by humans has increased by about 40% from 2000 to 2010 [1, 2]. As a consequence, the prevalence of antibiotic-resistant bacteria (AMRB) has increased worldwide, limiting our ability to fight infectious diseases [3–6], and resulting in a higher risk of morbidity from infections [7–9].

Antimicrobial resistance (AMR) has thus emerged as a major public health issue over the past decade [10]. Despite implementation of several national and global action plans, worldwide antibiotic consumption and AMR rates remain high [10–12]. In France, for example, 29% of *Klebsiella pneumoniae* were resistant to third-generation cephalosporins in 2016 [11]. According to the Centers for Disease Control and Prevention, infections due to resistant microorganisms were associated with 25 000 deaths in Europe in 2007 [13] and exceeded 23 000 deaths in the USA in 2013 [12]. A review on AMR commissioned by the UK Prime Minister estimated that 700 000 AMR-attributable deaths occurred globally in 2014 [14]. According to that study, the current rising AMR trend would cause 10 million deaths worldwide in 2050, thereby becoming the leading cause of mortality, with an annual mean of 390 000 deaths expected in Europe. The French Institute for Public Health estimated that, in 2012, 158 000 infections were caused by multidrug-resistant bacteria (MDRB) in France, and 12 500 deaths were associated with these infections [15].

Nationwide estimates of the overall burden of AMR [14–18] are however hampered by the lack of comprehensive and national data. Thus, most of the time surveillance data have been used to derive such estimates. However, surveillance reports focus on defined microorganism–antibiotic pairs, and collect selected data. The European Antimicrobial Resistance Surveillance Network (EARS-net), for example, collects data from local and clinical laboratories in 30 countries, but surveillance is limited to invasive infections and specific AMRB, and its coverage varies by country. It was estimated that EARS-Net covers 18% of hospital bed-days in France (except for *Streptococcus pneumoniae*, for which it was estimated at 67%) [11, 16]. Surveillance data have thus been extrapolated to the national level, using weighting from the literature. These predictive models require strong assumptions and use different sources, which have often their own specifications [19].

In 2014, the World Health Organization (WHO) revised its codification of infections and AMR within the 10th revision of the international classification of diseases (ICD-10) [20]. In France, specific codes were added in 2015 to the French ICD-10 version such as ‘U82.10’ (methicillin-resistant *Staphylococcus aureus*; MRSA) or ‘U83.70’ (emerging highly resistant bacteria), to allow codification of colonisation or infection with AMRB which pose specific management issues (i.e. isolation precautions or complex therapeutic management) as per current French recommendations. Those refinements provide an opportunity to use the French National Hospital Discharge Database (French acronym PMSI) and ICD-10 codes to estimate the actual incidence of AMR based on real nationwide data, while using limited assumptions and estimations.

This study was undertaken to estimate the incidence of AMR in French hospitals in 2016, using the PMSI database.

## Methods

### Data collection

The study population included all incident hospitalisations with an acute infection caused by *Streptococcus*, *Staphylococcus*, Enterobacteriaceae or other Gram-negative bacteria identified in 2016 from the PMSI.

The PMSI database covers all hospitalisations (also referred to as stays) in French publicly funded and private hospitals. Because this database is used for reimbursement purposes, each hospitalisation description is standardised, following the Technical Agency for Hospital Information (ATIH) recommendations. It contains ICD-10-coded hospital diagnoses, medical procedures performed during each stay and individual data such as age, sex and geographic area of residence. Diagnoses are coded as primary diagnosis (PD: condition requiring hospitalisation), related diagnosis (RD: adds information to PD) and significant associated diagnosis (SAD: complications and co-morbidities potentially affecting the course or cost of hospitalisation). Anonymised PMSI data from 2016 were extracted from the French National Health Data System (SNDS) that records every individual's demographic information, healthcare encounters and drug reimbursements [21]. We restricted the study to metropolitan France (henceforth referred to as France), excluding overseas territories, and representing 96% of its population in 2016, and hospitalisations in short-stay institutions (medicine, surgery, obstetrics).

Stays with admission between 1 January and 31 December 2016 were selected, when the PD, RD or SAD contained at least one specific infection code. Only stays longer than 1 day were considered as hospitalisation. To identify stays with acute infections, lists of ICD-10 codes corresponding to infections, microorganisms and resistance markers were established in collaboration with infectious diseases and PMSI coding specialists (Supplementary Tables S1 and S2). Codes only corresponding to colonisation with AMR were excluded from these lists. Most data available in the literature focuses on the first infection episode in a given year; in order to provide comparable data, only the first infectious episode in patients hospitalised within 2016 was selected. Because of the structure of the database and possible missing bacterial data, the first infectious episode was defined as the first infection that occurred at an anatomical site, also referred to as ‘incident infection’.

### Selection and recoding algorithm, and extrapolation

Using PMSI to estimate the incidence of AMR among hospitalised patients proved challenging. Three major constraints of the database were identified.

The first one is related to database structure. Indeed, in the database, diagnoses are not linked: infections are not linked with their causal bacteria or bacteria with their resistance status, except for some specific codes such as ‘J13’ (pneumonia due to *S. pneumoniae*) or ‘U82.10’ (MRSA) (Table S1). Therefore, linking an infection to its aetiological microorganism and its AMR status was not possible when several infections, pathogens and/or resistance statuses were coded for a given stay. Consequently, stays with several infection sites were excluded, except for blood infections and medical device infections. Indeed, stays with blood infection and another site recorded were categorised as infection of the recorded site. Likewise, medical device infection was favoured when associated with an infection at the same site, e.g. ‘T82.6’ (infection due to cardiac valve prosthesis) and ‘I33.0’ (infective endocarditis).

The second limitation is related to French coding practices and its clinical perspective. Indeed, in France, coding rules imply that AMR status is notified only when resistance implies changing usual clinical management. Consequently, natural resistance or resistance that does not result in modification of conventional management or therapeutic difficulties would not be recorded.

Finally, some stays with infection might not be associated with an infecting microorganism. No microorganism code may indicate a lack of microbiological testing (i.e. the physician treated the infection empirically); or it may indicate negative results of samplings, part of which may be secondary to effective antibiotic therapy administered before sampling.

**Fig. 1.**
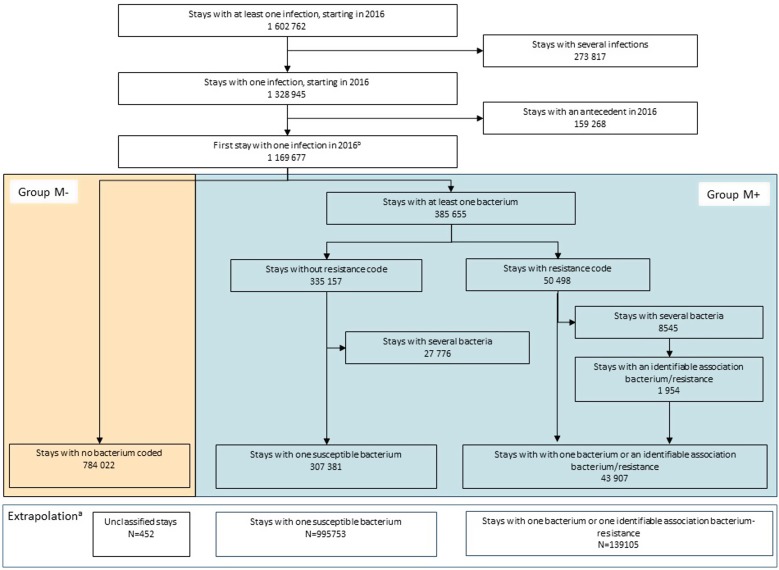
Flowchart of patient selection, group constitution and extrapolationa – France, 2016.

^a^*Group M*^−^ missing resistance status was extrapolated from group *M*^+^ susceptible and resistant bacteria, according to sex, age and site of infection and the variable described, listed in Table 2. Because of insufficient sample size, 452 stays could not be extrapolated. ^b^The first infectious episode in patients hospitalized within 2016 was defined as the first infection that occurred at an anatomical site.

From the first 2016 stay with one infection, two groups were defined: (*M+*) stays, with at least one microorganism coded; and (*M−*), with no microorganism coded (Fig. 1). For *M+*, to minimise the number of excluded stays with multiple bacteria codes and only one specific AMR code recorded, resistance was attributed to the most relevant bacteria coded, excluding the other bacterial codes (e.g. methicillin resistance was assigned to *S. aureus*, ESBL resistance to *Enterobacteriaceae* or other Gram-negative bacteria and vancomycin resistance to *Enterococcus*). If attribution was not possible, stays with several microorganisms were excluded. In addition, to minimise inappropriate AMR categorisation (e.g. natural resistance), non-concordant microorganism–resistance pairs were recoded to the nearest most relevant (e.g. penicillin-resistant *Enterobacteriaceae* as ESBL, and vancomycin-resistant *Streptococcus* as *Enterococcus*). Relevant microorganism–resistance pairs are listed in Supplementary Table S2. Finally, when assignment to a given microorganism was not possible because of several resistance codes or impossible recoding, AMR status was retained but resistance was classified as ‘unknown’.

Given the above limitation and the large number of stays with missing bacteria codes, we first analysed stays with complete information, i.e. those with an infection associated with an identified microorganism, whether resistant or susceptible. Extrapolation was then performed on the *M−* group, assuming that these stays were similar to group *M+*, conditional on gender, age and infection site stratum (classified as below). Thus, microorganism and AMR distributions were extrapolated from group *M+*, according to those parameters. For descriptive purposes, resistance was also extrapolated according to some patients’ and stay recorded characteristics: Charlson comorbidity index, infection as PD, length of stay, surgical procedure during the stay and in-hospital death. Consequently, some minor resistance-distribution differences could be noted, reflecting marginal differences in distributions of these variables between the *M+* and *M−* groups. When <10 *M+* stays were found for a given set of gender, age class and infection site, extrapolation was not performed and the corresponding observations were excluded. 95% CI were estimated for extrapolated number of stays and resistance rates. Moreover, the distributions of the different categories within a variable were calculated from the estimated number of stays. Since confidence interval were defined independently for each class, it was thus not relevant to estimate confidence interval for distributions.

### Variables

Patients were characterised by gender, age (<28 days, 28 days–5 years, 6–15, 16–35, 36–65, 66–80, >80) and Charlson comorbidity index (0, 1–2, 3 or >4) [22].

Stays were characterised by their duration (1–7, 7–14 and >14 days), characteristic of infection (PD or not), surgical procedure during the stay (yes or no) and in-hospital mortality. Surgical procedures were identified by their codes on the French common classification of medical procedures [23]. In-hospital mortality was recorded if the patient died during hospitalisation, regardless of the cause of death.

The following variables were used to describe the infections: site and bacterial species involved, AMR status and resistance type. Infection sites were stratified into 13 categories (Table S1): (1) urinary and genital tracts, (2) medical device associated, (3) skin and soft tissues, (4) lower respiratory tract, (5) abdomen and digestive tract, (6) bacteraemia and sepsis (henceforth blood infection), (7) infection during pregnancy, (8) bone and joint, (9) newborn infection, (10) heart and mediastinum, (11) ear, nose and throat, (12) eye, (13) nervous system. Medical device infections included foreign body infections and those associated with diagnostic procedures. Bacterial species were classified as *Staphylococcus*, *Streptococcus*, Enterobacteriaceae or other Gram-negative bacteria and by species. Resistance was categorised as to (1) penicillin, (2) methicillin, (3) extended-spectrum *β*-lactamase (ESBL), (4) other *β*-lactams resistance mechanisms, (5) vancomycin and related, (6) quinolones, (7) multiple antibiotics, (8) highly drug-resistant, (9) MDRB, (10) other or unspecified or (11) unknown (as explained above). In the French coding system, a MDRB code means that ‘multidrug-resistant’ is written in the bacteriology reports; conversely ‘resistant to multiple antibiotics’ is used when the bacteria is resistant to several antibiotics but multidrug-resistant is not written. It was assumed that MDRB refers to a bacterium resistant to at least three classes of antibiotic to which it is normally sensitive. Some specific resistances cannot currently be extracted from the PMSI, for example, resistance to third-generation cephalosporins is coded within group [4]. Microorganisms were considered susceptible to antibiotics when no resistance was coded (i.e. there was indeed no resistance marker detected, or if present, the resistance was not clinically significant and did not require a modification of conventional management of infection).

### Analyses

To validate *M+* selection and reclassification, *S. aureus*, *Enterobacteriaceae* and *Streptococcus* estimated resistance rates were compared with those available from 2016 EARS-net data for France [11]. Rates were estimated from *M+* blood infections (primary and secondary) and meningitis (*Enterobacteriaceae and S. pneumoniae* only) in order to be compatible with EARS-net data. EARS-net ESBL-pE rates were estimated from rates provided for third-generation cephalosporin-resistant isolates [11, 24]. The same validation was conducted on the extrapolated population.

The overall population was described after extrapolation. The characteristics of patients (gender, age and Charlson index), stays (infection as PD, duration, surgery during the stay, in-hospital mortality) and infections (site) were studied. For each infection site, resistance rates were estimated. Microorganism distribution in the population, associated resistance rates and frequencies of the most frequent bacterium–resistance pairs were studied. Because of the very large size of the sample, and the wide variety of hospitalisations and infections, no statistical test was used to compare the two groups of patients having resistant or susceptible bacterial infections.

Finally, incidences of total AMRB and of the most frequent bacterium–resistance pairs were estimated, by dividing the number of hospitalisations with AMRB infection by the total number of days of hospitalisations in short-stay institutions (medicine, surgery, obstetrics), in metropolitan France in 2016 [25]. They were expressed as the number of cases per 1000 patient-days.

Statistical analyses were computed with SAS Enterprise Guide (*v* 7.13 software, SAS Institute Inc., Cary, NC, USA). The study and data extraction were approved by the French Data Protection Agency (CNIL, approval DE-2016–176).

## Results

In 2016, among 1 602 762 infection-associated stays, 70.8% were retained for analysis, corresponding to 1 135 310 first stays without prior infection at the same site in the corresponding year (Fig. 1). During the selection process, 19.2% hospitalisations were excluded because of several infection or bacterial codes. Bacteria were coded in 30.9% of the selected sample (*group M+*), 12.5% of which were antibiotic-resistant, with 4.7% of resistance codes attributed to one of several bacteria, 20.3% of bacterium–resistance pairs recoded and 20.1% of resistances classified as ‘unknown’ (15.2% with inconsistent bacterium–resistance pairs and 4.9% with several resistance codes). Extrapolation yielded a total of 139 105 (95% CI 127 920–150 289) stays associated with an AMR infection, corresponding to 12.3% (95% CI 11.3–13.2) of the sample. Since extrapolation was not performed when the matching subgroup sample was deemed too small (<10 stays), a total of 452 stays were excluded from the analyses.

### Validations

Resistance rates estimated from *M+* blood infections and meningitis were in accordance with those provided by EARS-net (Table 1). After using the recoding algorithm, 14.3% (95% CI 13.6–15.0) of *S. aureus* were resistant to methicillin, 0.5% (95% CI 0.3–0.7) *Enterococcus* were resistant to vancomycin and 0.3% (95% CI 0.1–0.5) *K. pneumoniae* were resistant to carbapenem in the study sample, similar to EARS-net France results. In the same way, the percentage of ESBL resistance among *K. pneumoniae* (19.6%; 95% CI 18.4–20.8) *and E. coli* (11.6%; 95% CI 11.2–12.0) were close to the EARS-net estimations, while *S. pneumoniae* resistance rates were slightly above EARS-net estimations.

**Table 1.** Rates of MRSA, ESBL-p *Enterobacteriaceae*, emerging highly drug-resistant^a^ bacteria and penicillin-resistant *Streptococcus pneumoniae* among blood and cerebrospinal sampling, and comparison with EARS-Net^b^ surveillance data, France 2016

	Before extrapolation^c^, selected sample			After extrapolation^c^, whole cohort		EARS-net 2016
	Crude data^d^	Modified sample^d^				
	Rates of resistance	Number of isolates	Rates of resistance	Number of isolates	Rates of resistance	Rates of resistance
*Staphylococcus aureus* (methicillin)	10.8	10 840	14.3 (13.6–15.0)	27 613	14.1 (11.0–17.2)	13.8
*Enterococcus* (vancomycin)	0.2	3789	0.5 (0.3–0.7)	8571	0.6 (0.0–1.4)	0.6
*Streptococcus pneumoniae* (penicillin)	0.7	3083	0.8 (0.5–1.1)	11 291	0.7 (0.0–1.5)	0.1
*Escherichia coli* (ESBL-p)^e^	5.9	29 862	11.6 (11.2–12.0)	55 333	11.7 (10.0─13.3)	8.3
*Klebsiella pneumoniae* (ESBL-p)^e^	13.6	4345	19.6 (18.4–20.8)	9753	18.5 (13.1–23.9)	20.2
*Klebsiella pneumoniae* (carbapenem)	0.3	4345	0.3 (0.1–0.5)	9753	0.4 (0.0–0.8)	0.4

a Emerging highly drug-resistant bacteria: vancomycin-resistant *Enterococcus* (VRE) and carbapenem-resistant *Klebsiella pneumoniae*.

b EARS-net: European Antimicrobial Resistance Surveillance Network [11]; blood infection: primary and secondary blood infection. Resistant *Enterobacteriaceae and Streptococcus pneumoniae* rates were estimated among blood and cerebrospinal sampling, while MRSA and VRE rates were estimated among blood sampling only, in order to be in accordance with EARS-net data.

c G*roup M^−^* missing resistance status was extrapolated from *group M^+^* susceptible and resistant bacteria, according to sex, age and site of infection. Because of insufficient sample size, 452 stays could not be extrapolated.

d In modified data, some stays with several codes of infection and bacteria were excluded. Some resistance codes were reclassified. In crude data, no modification in the database was made.

e EARS-Net ESBL-producing *Enterobacteriaceae* (ESBL-p) rates were calculated from 2016 rates of third-generation cephalosporin resistance and 2017 rates of ESBL-p among C3G-resistant isolates [11, 24].

MRSA, methicillin-resistant *Staphylococcus aureus*; ESLB-p E, ESLB-producing Enterobacteriaceae.

Likewise, estimates of resistance rates recorded from the extrapolated sample were consistent with those provided by EARS-net for MRSA, the emerging highly drug-resistant bacteria, ESBL-p *E. coli* and *K. pneumoniae* as well as for penicillin-resistant *S. pneumoniae* (Table 1).

### Patient and stays

Around 58% of patients infected with AMRB were >65 years old and >20% had a Charlson index >2, as compared with 52% and 16% of those infected with a susceptible strain, respectively (Table 2). Infection was the PD for slightly over half of stays in both groups; 39% of patients with an infection with AMRB underwent surgery during hospitalisation, as compared with only 29% of those infected with a susceptible strain. Over 20% of the stays with AMRB exceeded 2 weeks, in contrast with <15% of stays without AMRB. Death occurred during 7.4% of the stays with an infection with AMRB, corresponding to 17.6% of in-hospital deaths.

**Table 2.** Patients, incident hospital stays and infection characteristics by resistance status on the whole cohort, after extrapolation^a^ – France, 2016 (*n* = 1 134 858)

Patient characteristics	Resistant		Susceptible		Percentage of resistance
	*n* (95% CI)	%	*n* (95% CI)	%	% (95% CI)
Sex					
Male	70 531 (65 067–75 994)	50.7	458 947 (453 484–464 411)	46.1	
Female	68 574 (62 853–74 295)	49.3	536 806 (531 085–542 527)	53.9	
Age (years)					
<15 years	10 092 (8125–12 053)	7.3	114 671 (112 710–116 638)	11.5	
15–35 years	13 632 (11 742–15 525)	9.8	140 533 (138 640–142 423)	14.1	
36–65 years	34 977 (32 501–37 453)	25.1	220 395 (218 259–222 529)	22.1	
66–80 years	35 983 (33 849–38 119)	25.9	245 492 (243 016–247 968)	24.7	
>80 years	44 421 (41 703–47 139)	31.9	274 662 (271 944–277 380)	27.6	
Charlson comorbidity index^b^					
0	64 687 (58 177–71 187)	46.8	551 250 (544 825–557 835)	55.3	
1–2	44 417 (41 497–47 336)	32.2	283 402 (280 466–286 305)	28.4	
3–4	17 218 (16 253–1818)	12.4	95 144 (94 107–96 035)	9.5	
⩾5	11 767 (11 152–12 379)	8.5	66 973 (66 245–67 472)	6.7	
Stay characteristics					
Infection as primary diagnosis^b^	74 726 (67 852–81 589)	53.7	525 477 (51 858–532 322)	52.8	
Length of stay^b^					
<7 days	62 788 (56 175–69 401)	47.6	576 314 (569 672–582 898)	57.5	
7–14 days	40 238 (37 634–41 842)	30.5	280 615 (278 013–283 221)	28.0	
>14 days	28 904 (144 614–27 537)	21.9	146 003 (144 614–147 351)	14.6	
Surgical procedure during the stay^b^	49 963 (45 862–54 073)	39.0	272 601 (268 477–276 688)	29.0	
In-hospital death^b^	10 184 (9629–10 735)	7.4	47 640 (48 148–47 042)	4.8	
Infection characteristics					
Infection site					
Urinary and genital tract	34 012 (33 554–34 469)	24.4	226 826 (226 369−227 284)	22.8	13.0 (12.9–13.2)
Lower respiratory tract	31 775 (28 928–34 619)	22.8	263 175 (260 331–266 022)	26.4	10.8 (9.8–11.7)
Gastrointestinal and abdominal	24 293 (21 524–27 058)	17.5	164 291 (161 526–167 060)	16.5	12.9 (11.4–14.3)
Skin and soft tissues	19 843 (18 132–21 556)	14.3	87 278 (85 565–88 989)	8.8	18.5 (16.9–20.1)
Material infection	10 096 (9840–10 355)	7.3	41 902 (41 643–42 158)	4.2	19.4 (18.9–19.9)
Primary blood infection^c^	9900 (8996–10 805)	7.1	65 844 (64 939–66 748)	6.6	13.1 (11.9–14.3)
Ear, nose and throat	1995 (1138–2851)	1.4	45 784 (44 928–46 641)	4.6	4.2 (2.4–6.0)
Heart and mediastinum	2861 (2151–3571)	2.1	20 040 (19 330–20 750)	2.0	12.5 (9.4–15.6)
Bone and joint	2052 (1802–2305)	1.5	10 202 (9949–10 452)	1.0	16.7 (14.7–18.8)
Infection during pregnancy	1047 (1007–1088)	0.7	43 948 (43 907–43 988)	4.4	2.3 (2.2–2.4)
Infection in newborn	638 (588–688)	0.5	19 457 (19 407–19 507)	1.9	3.2 (2.9–3.4)
Eye	437 (213–661)	0.3	4019 (3795–4243)	0.4	9.8 (4.8–14.8)
Nervous system	156 (47–263)	0.1	2987 (2880–3096)	0.3	5.0 (1.5–8.4)

a G*roup M^−^* missing resistance status was extrapolated from *group M^+^* susceptible and resistant bacteria, according to sex, age and site of infection and each of the variable considered. Because of insufficient size of the matched sample per subgroup, 452 stays could not be extrapolated.

b The resistance status was extrapolated according to sex, age, site of infection and the variable considered. Consequently, the number of stays with resistance may vary depending on the variable.

c Bacteraemia or sepsis, not associated with another site of infection.

### Infection characteristics

#### Site

Infections with an AMRB were mostly urinary and genital, lower respiratory tract, gastrointestinal and abdominal, and skin and soft tissues infections (24.4%, 22.8%, 17.5% and 14.3%, respectively) (Table 2). Except for lower respiratory tract infections, the most common infections were associated with high resistance rates, with AMR rates equal to: 13.0% (95% CI 12.9–13.2) (urinary and genital tract), 12.9% (95% CI 11.4–14.3) (gastrointestinal and abdominal), 18.5% (95% CI 16.9–20.1) (skin and soft tissues), 19.4% (95% CI 18.9–19.9) (medical device) and 13.1% (95% CI 11.9–14.2) (blood infections). For the remaining 7% of infections, high AMR rates were observed for bone and joint (16.7%; 95% CI 14.7–18.8) and heart and mediastinum (12.5%; 95% CI 9.4–15.6) infections, which represented each <2% of the total infections with AMRB.

#### Microorganisms

Antibiotic-resistant microorganisms were mostly Enterobacteriaceae (49.9%) and *Staphylococcus* (33.5%) (Table 3). Enterobacteriaceae were predominantly *E. coli* (76.5%), followed by *K. pneumoniae* (17.0%), *Citrobacter* was uncommon (around 2% of Enterobacteriaceae), but often resistant. Resistant *Staphylococci* were mostly *S. aureus* (70.7%), the microorganism with the highest resistance rate (25.8%; 95% CI 21.4–30.2). Streptococci and the other Gram-negative bacteria accounted for <17% of resistant bacteria. Among them, only *Acinetobacter* had an average AMR rate similar to the overall mean rate (12.3% *vs* 12.2%, respectively).

**Table 3.** Distribution of micro-organisms by resistant status and percentage of resistance resulting from extrapolation^a^ – France, 2016 (*n* = 1 134 858)

	Resistant		Susceptible		Percentage of resistance^b^
	*n* (95% CI)	%	*n* (95% CI)	%	% (95% CI)
*Enterobacteriaceae*	69 488 (59 099–79 886)		387 835 (365 589–410 075)		15.2 (12.9–17.5)
*Escherichia coli*	53 162 (47 766–58 554)	76.5	315 198 (304 422–325 976)	81.3	14.4 (13.0–15.9)
*Klebsiella pneumoniae*	11 779 (8978–14 586)	16.9	39 543 (34 483–44 598)	10.2	22.9 (17.5–28.4)
*Proteus mirabilis*	2787 (1638–3945)	4.0	23 152 (19 691–26 611)	6.0	10.7 (6.3–15.2)
*Citrobacter*	1691 (754–2627)	2.4	8106 (5985–10 227)	2.1	17.3 (7.7–26.8)
*Yersinia*	69 (37–174)	0.1	1822 (1006–2637)	0.5	3.6 (0–9.2)
Unspecified	0	0	14 (2–26)	0.00	0.00
*Staphylococcus*	46 630 (36 580–56 699)		177 032 (155 004–199 051)		20.8 (16.3–25.3)
*Staphylococcus aureus*	32 980 (27 321–38 651)	70.7	94 916 (85 214–104 603)	53.6	25.8 (21.4–30.2)
Other	10 599 (7573–13 624)	22.7	56 381 (48 744–64 020)	31.8	15.8 (11.3–20.3)
Unspecified	3051 (1686–4424)	6.6	25 735 (21 046–30 428)	14.5	10.6 (5.8–15.4)
*Streptococcus*	8756 (3752–13 738)		261 975 (234 310–289 650)		3.2 (1.4–5.1)
*Enterococcus*	3464 (1999–4915)	39.6	34 910 (30 116–39 709)	13.3	9.0 (8.3–9.7)
Other	3329 (1350–5302)	38.0	130 844 (120 524–141 159)	49.9	2.5 (1.0–3.9)
Unspecified	647 (11–1282)	7.4	24 247 (18 678–29 821)	9.3	2.6 (0.1–5.1)
*Streptococcus pneumoniae*	1316 (392–2239)	15.0	71 974 (64 992–78 961)	27.5	1.8 (0.5–3.0)
Other Gram-negative bacteria	14 224 (8104–20 340)		168 918 (146 566–191 251)		7.8 (4.4–11.1)
*Pseudomonas aeruginosa*	8024 (5198–10 850)	56.4	65 717 (57 667–73 739)	38.9	10.9 (7.0–14.7)
*Haemophilus influenzae*	1835 (795–2873)	12.9	41 833 (36 225–47 439)	24.8	4.2 (1.8–6.6)
*Acinetobacter*	524 (46–1001)	3.7	3733 (1931–5543)	2.2	12.3 (1.1–23.5)
*Campylobacter*	1554 (807–2301)	10.9	37 279 (34 037–40 520)	22.1	4.0 (2.1–5.9)
Other	2287 (1258–3315)	16.1	20 356 (1670–24 010)	12.0	9.0 (5.2–12.8)
Total	139 098 (129 354–148 860)		995 730 (985 998–1 005 504)		12.2 (11.4–13.1)

a G*roup M*^*−*^ *m*issing microorganisms and resistance status were extrapolated from *group M^+^* bacteria, according to sex, age and site of infection. Because of insufficient sample size, 452 stays could not be extrapolated.

b Percentage of antibiotic-resistant isolates within each species or group.

MRSA were principally isolated from lower respiratory tract (32.3%), skin and soft tissues (31.3%) and primary blood infections (8.7%) (Table 4). ESBL-p Enterobacteriaceae were mostly identified in urinary and genital tract, gastrointestinal and lower respiratory tract infections (respectively, 43.6%, 28.3%, 12.2% for *E. coli* and 32.3%, 13.8% and 26.9% for *K. pneumoniae*). More than a third of carbapenem-resistant *K. pneumoniae* was identified in lower respiratory tract, and around 20% in urinary and genital tract infections. Half of the vancomycin-resistant *Enterococcus* was associated with gastrointestinal infections. Finally, around 76% of penicillin-resistant *S. pneumoniae* were identified in lower respiratory tract.

**Table 4.** Distribution of the infection sites with frequency >5%, for the main microorganism–resistance pairs – France, 2016 (*n* = 1 134 858)

	*N*	Per cent (%)
MRSA		
Lower respiratory tract	6289 (4982–7596)	32.3
Skin and soft tissues	6099 (5110–7088)	31.3
Primary blood infection^a^	1699 (1334–2064)	8.7
Material infection	1531 (1425–1638)	7.8
Urinary and genital tract	1060 (990–1131)	5.4
ESBL-p *Escherichia coli*		
Urinary and genital tract	15 801 (15 480–16 122)	43.6
Gastrointestinal and abdominal	10 247 (8370–12 123)	28.3
Lower respiratory tract	4405 (3406–5405)	12.2
Sepsis	1839 (1484–2193)	5.1
ESBL-p *Klebsiella pneumoniae*		
Urinary and genital tract	2769 (2637–2901)	32.3
Lower respiratory tract	2307 (1544–3070)	26.9
Gastrointestinal and abdominal	1184 (593–1775)	13.8
Primary blood infection^a^	688 (456−920)	8.0
Skin and soft tissues	678 (388−968)	7.9
Material infection	638 (567–708)	7.4
Carbapenem-resistant *Klebsiella pneumoniae*		
Lower respiratory tract	61 (0–153)	39.8
Urinary and genital tract	31 (019–44)	20.3
Skin and soft tissues	20 (0–53)	13.4
Primary blood infection^a^	17 (0–42)	11.3
Vancomycin-resistant *Enterococcus*		
Gastrointestinal and abdominal	123 (0–278)	51.1
Skin and soft tissues	32 (0–86)	13.2
Ear, nose and throat	22 (0–59)	8.9
Heart and mediastinum	18 (0–49)	7.4
Primary blood infection^a^	17 (0–43)	7.1
Urinary and genital tract	17 (0–26)	6.9
Penicillin-resistant *Streptococcus pneumoniae*		
Lower respiratory tract	373 (92–655)	75.9
Ear, nose and throat	66 (0–155)	13.4
Primary blood infection^a^	28 (0–58)	5.7

a Bacteraemia or sepsis, not associated with another site of infection.

MRSA, methicillin-resistant *Staphylococcus aureus*; ESBL-p: extended spectrum *β*-lactamase producing bacteria.

**Fig. 2.**
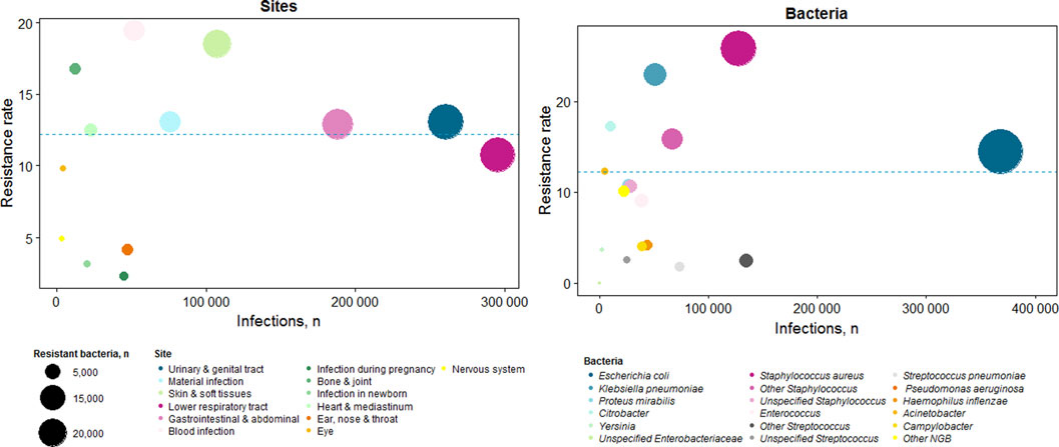
Distribution of infection sites and microorganisms according to number of stays and resistance rate^a^ – France, 2016 (*n* = 1 134 858). Each colour represents an infection site or a bacteria, depending on the graph. The size of the circle represents the number of resistant infection. The total number of infections (with or without resistant bacteria) is presented on x-axis, and the percentage of resistance on y-axis.

^a^Resistance rate: all type of resistance were considered.

Overall, the three most frequent resistant bacteria populations were *E. coli* (38.2%), *S. aureus* (23.7%) and *K. pneumoniae* (8.5%) (Fig. 2). Among stays with resistance, 68% of *E. coli* were ESBL-p, 59% of *S. aureus* were methicillin-resistant and 72% of *K. pneumoniae* were ESBL-p. Therefore, the most frequent resistant pathogens were ESBL-p *E. coli* (26.0%), MRSA (14.0%) and ESBL-p *K. pneumoniae* (7.2%).

In 2016, infections associated with AMRB represented 139 105 (95% CI 127 920–150 289) stays. MRSA caused 19 493 (95% CI 15 237–23 747) infections, and ESBL-p Enterobacteriaceae, 49 692 (95% CI 47 223–52 142), including 36 195 (95% CI 31 734–40 655) infections caused by ESBL-p *E. coli* and 8566 (95% CI 6167–10 965) by *K. pneumoniae*. The overall AMRB incidence was thus estimated at 2.52 (95% CI 2.31–2.72) hospitalisations per 1000 patients-days. Infections caused by MRSA represented 0.35 (95% CI 0.27–0.42) hospitalisations per 1000 patients-days and ESBL-p Enterobacteriaceae 0.89 (95% CI 0.85–0.94), including 0.65 (95% CI 0.57–0.74) for *E. coli* and 0.15 (95% CI 0.11–0.19) for *K. pneumonia.*

## Discussion

This innovative real-life study used an administrative medical database to assess the nationwide AMR incidence in France. It was estimated that around 140 000 (95% CI 130 000–150 000) hospitalisations were associated with an infection due to AMRB in 2016. The PMSI database provides a number of information allowing detailed description of infections and associated stays. However, our analysis also emphasises the difficulties encountered when using such data sources for an objective other than their original purpose, particularly when analysing infections and AMR. Indeed, contrarily to the majority of studies in other areas where simply identifying the diagnosis of a given disease is sufficient, studies dealing with microbial resistance require identifying both the infection and the associated microorganism responsible for the infection, as well as the potentially associated resistance marker.

One of the strengths of this database is that it covers all hospital stays in France. Administrative databases are increasingly used for epidemiological and diseases burden studies in various countries, but they have not yet been used to measure the AMR burden [26–29]. They provide access to ‘real-life’ data of the national population, and recent works illustrate the value of such data, particularly in the field of infectious diseases [29–33]. Sahli *et al*. concluded that quality of coding of infection diagnoses in the PMSI was high for PD, with positive predictive values of correct coding ranging between 0.98 (95% CI 0.95–1.00) and 0.93 (95% CI 0.88–0.98), but inferior for RD 0.70 (95% CI 0.61–0.71) [31]. Moreover, Afshar *et al*. considered that ICD-10 codes would enable analyses of different pathogens and their drug-resistance patterns [33].

However, the database refund-management purposes led to some difficulties. Indeed, hospital reimbursements are determined by diagnosis, concomitant diseases and stay duration, directly extracted from PMSI. Consequently, concomitant events at diagnosis or complicating the disease are well encoded but information not used for reimbursement calculation could be absent or miscoded. Thus, first, a multi-level algorithm of selection and recoding was required, developed in association with infectious disease specialists and public health doctors specialised in PMSI-coding. Two Ph.D. students independently programmed the algorithm to verify it. For example, 35% of bacterium–resistance pairs were inconsistent in 2016, including natural resistance, inconsistent association between a given bacterium and a resistance marker, or resistance that would not usually influence management; 57% of them could be recoded. Moreover, 19% of stays were excluded because diagnostic codes were not linkable. Although recovery through resistance codes could induce a selection bias, thereby increasing the per cent of resistance, it corresponded to only 0.2% of the selected population and 0.7% of the stays excluded. Conversely, exclusion of stays with multiple codes could cause underestimation of AMR rates and bias stays descriptions: 16% of them were associated with at least one resistance code, and hospitalisations were more severe (longer stays, higher in-hospital mortality, higher Charlson score, older patients, data not shown). Finally, because around 70% of stays had no bacteria coded, an extrapolation was conducted, assuming that, conditioning on gender, age and infection site, stays with missing bacteria code had similar profiles to those with available information. An alternative approach to extrapolation was tested for the *M−* group, where all missing microorganisms were assumed to be susceptible (data not shown). However, resulting resistance rates were far below surveillance and literature data.

Database recoding and extrapolation were validated, comparing our study results with surveillance data from EARS-net [11]. Moreover, our incidence estimates were consistent with other 2016 French data, estimating MRSA and ESBL-p *Enterobacteriaceae* incidence to 0.34 and 0.95 hospitalisations per 1000 patients-days, in short-stay institutions [34]. This study estimated incidence from the first 3 months of 2016, from 1354 health institutions (short-stay, psychiatry, long-term care facilities…). In addition, our study estimates were consistent with the low range of the MDRB-infection estimate of 158 000 (127 000–245 000) generated for France in 2012 [15]. Several factors might explain our relatively low estimates. First, the latter study was based on 2012 data and used prevalence estimations whereas our study focused on first hospitalisations with acute infections and 2016 hospitalisation data. Moreover, most of the patients with several infections or bacteria were excluded (with 16% resistance), notably the most serious infections. Finally, in the PMSI, AMR should be coded only if it alters clinical management and rates can be strongly influenced by clinical practices. Indeed, if microbiological investigations are not performed or impractical and first-line treatments include broad-spectrum antibiotics, AMR is unlikely to alter clinical management. For example, urinary and genital infections were more frequently associated with bacteria codes, which can be explained by commonly obtained urine cultures in hospitals, unlike for example, bronchopulmonary secretions samplings. The distribution of infection sites among stays without coded microorganisms supports this hypothesis, with a higher proportion of lower respiratory tract and abdominal and gastrointestinal infections (Supplementary Table S3).

In this study, patients infected with AMRB were slightly older and had more comorbidities than the others. They underwent a surgical procedure more often during hospitalisation, and their length of stay was longer. Even though not attributable to AMRB, in-hospital death occurred more often during hospitalisation with an infection with ARMB than in those without ARMB.

In conclusion, estimating the AMR incidence based on hospital discharge database requires resolving complex problems in data handling, necessitating hypotheses to correct for missing or inconsistent ICD-10 codes. However, the resulting information, consistent with data derived from surveillance studies, is very thorough, indeed covering all acute bacterial infections and a variety of selected microorganisms, thereby providing more comprehensive information than most available estimates based on a microorganism or disease selection.

## Supplementary material

For supplementary material accompanying this paper visit https://doi.org/10.1017/S0950268819000402. click here to view supplementary material
