# Supplementary material for: Hospitalisations with infections related to antimicrobial-resistant bacteria from the French nationwide hospital discharge database, 2016
Source: Epidemiol Infect. 2019 Mar 12;147:e144. doi: 10.1017/S0950268819000402 (PMC6518510; doi:10.1017/S0950268819000402)
Supplement: Supplementary file 1 [file S0950268819000402sup001.docx]

Epidemiology and Infection: **Incident hospitalizations with infections related to antimicrobial resistant bacteria from the nationwide hospital discharge database, France 2016**

M. OPATOWSKI, P. TUPPIN, K. COSKER, M. TOUAT, G. DE LAGASNERIE, D. GUILLEMOT, J. SALOMON, C. BRUN-BUISSON, L. WATIER

Supplementary Material

Supplementary Table S1: ICD-10 codes used for selection and classification of infections, microorganisms and resistance markers

|  | ICD-10 codes |
| --- | --- |
| Infection site |  |
| Urinary & genital tracts | N10, N11.0, N11.1, N12, N13.6, N15.1, N30.0, N30.9, N34, N34.0-N34.2, N39.0, N41.0-N41.3, N45.0, N45.9, N70.0, N70.9, N73.0, N73.2, N73.3, N73.5, N75.1, N76.0, N76.2, N76.4 |
| Associated with medical devices^a^ | T80.2, T81.1, T81.4, T82.6, T82.7, T83.5, T83.6, T84.5-T84.7, T85.7 |
| Skin & soft tissues | A46, A48.0, J34.0, L02-L04.9, L05.0, L08.8, L08.9, L73.2, L88, L89-L89.9^b^, L97^b^, M60, M60.0-L60.009, M65.0-M65.09, M71.0-M70.09, M71.1-M71.9, M72.6-M72.69, R02, T31-T31.9^b^, T79.3 |
| Lower respiratory tract | J13-J15.9, J16.8, J17.0, J17.8, J18-J18.9, J20-J20.2, J20.8 , J20.9, J22, J85.0-J85.2, J90^b^ |
| Abdomen & digestive tract | A04.0-A04.8, K35.0-K35.9, K36, K37, K40.1, K40.4, K41.1, K41.4, K42.1, K43.1, K43.4, K43.7, K44.1, K45.1, K57.0, K57.2, K57.4, K57.8, K61.0-K61.4, K63.0, K65.0-K65.9, K75.0, K80.0, K80.1, K80.3, K80.4, K81.0, K81.8, K81.9, K83-K83.3^b^, K83.5-K83.9^b^, D73.3 |
| Blood ^c^ | A400-A419, R57.2, R65.0, R65.1 |
| Infection during pregnancy | O23-O23.9, O41.1, O85, O86, O86.0, 086.1, O86.2, O86.3, O86.8, O88.3, O91.1 |
| Bone and joint | M00-M00.99, M01.3-M01.39, M01.8-M01.89^b^, M46.2- M46.59, M49.1-M49.39^b^, M860-M86.29, M86.8-M86.99, Z76.800 |
| Newborn infection | P00.2^b^, P23.2-P23.8, P36-P36.99, P39.0-P39.4 |
| Heart & mediastinum | I30.1, I30.9, I32.0, I33.0, I33.9, I40.0, I70.01, I70.21, I70.81, I70.91, J85, J85.3, J86.0, J86.9, J98.5 |
| Ear, nose and throat | H60, H60.0, H60.1, H60.3, H62, H62.0, H66, H66.0, H66.4, H67, H67.0, H67.8, H68, H68.0, H70, H70.0, H70.2, H70.9, J01-J06.8, J36, J39.0, J39.1, K12.2 |
| Eye | H13.1^b^, H13.2, H15, H15.0, H15.1, H16, H16.0^b^, H16.1-H16.9, H30-H30.9 |
| Nervous system | G00-G00.9, G01, G03.9, G04.2, G05.0, G06-G06.2, G07 |
| Micro-organism |  |
| *Streptococcus* | A40.0-A40.9, A49.1, B95.0-B95.5, G00.1, G00.2, J02.0, J13, J15.3, J15.4, J20.2, M00.1-M00.29, P23.3, P36.0-P36.19 |
| *Streptococcus pneumoniae* | A40.3, B95.3, G00.1, J13, M00.1-M00.109 |
| *Enterococcus* | A40.2, B95.2 |
| Other | A40.0, A40.1, B95.0, B95.1, B95.4, J02.0, J15.3, J15.4, P23.3, P36.0-P36.09 |
| Unspecified | A40.8, A40.9, A49.1, B95.5, G00.2, J20.2, M00.2-M00.29, P36.1-P36.19 |
| *Staphylococcus* | A41.0-A41.2, A49.0, B95.6, B95.7, B95.8, G00.3, J15.2, M00.0-M00.09, P23.2, P36.2-P36.29, P36.3-P36.39, U82.10^d^, U82.100^d^, U82.10+0^d^, U82.18^d^, U82.180^d^, U82.18+0^d^ |
| *Staphylococcus aureus* | A41.0, B95.6, P36.2-P36.29, U82.10^d^, U82.100^d^, U82.10+1^d^ |
| Other | A41.1, B95.7  = |
| Unspecified | A41.2, A49.0, B95.8, G00.3, J15.2, M00.0-M00.09, P23.2, P36.3-P63.9, U82.18^d^, U82.180^d^, U82.18+0^d^ |
| *Enterobacteriaceae* | A04.0-A04.4, A04.6, B96.1, B96.2, B96.4, B96.81, J15.0, J15.5, M49.2-M49.29, P23.4, P36.4 |
| *Escherichia coli* | A04.0-A04.4, B96.2, J15.5, P23.4, P36.4-P36.49 |
| *Klebsiella pneumoniae* | B96.1, J15.0 |
| *Proteus mirabilis* | B96.4 |
| *Citrobacter* | B96.81 |
| *Yersinia* | A04.6 |
| Unspecified | M492-M49.9 |
| Other Gram negative bacteria | A04.5, A41.3, A49.2, B96.3, B96.5, B96.80, G00.0, J14, J15.1, J20.1, M49.1-M49.19, P23.5 |
| *Pseudomonas aeruginosa* | B96.5, J15.1, P23.5 |
| *Acinetobacter* | B96.80 |
| *Haemophilus influenzae* | A41.3, A49.2, B96.3, G00.0, J14, J20.1 |
| *Brucella* | M49.1-M49.19 |
| *Campylobacter* | A04.5 |
| Resistance^d^ |  |
| Penicillin | U82.0, U82.0+0^e^, U82.00^e^ |
| Methicillin | U82.1-U82.18^e^, U82.100^e^, U82.10+0^e^, U82.180^e^, U82.18+0^e^ |
| Extended Spectrum β-Lactamase-producing | U82.2, U82.20^e^, U82.2+0^e^ |
| Other β-lactam resistance mechanims | U82.8, U82.80^e^, U82.8+0^e^, U82.9, U82.9+0^e^, U82.90^e^ |
| Vancomycin and related | U83.0, U83.00^e^, U83.0+0^e^, U83.1, U83.10^e^, U83.1+0^e^ |
| Quinolone | U83.2, U83.20^e^, U83.2+0^e^ |
| Multiple antibiotics | U83.7, U83.78^e^, U83.780^e^, U83.78+0^e^ |
| Emerging highly *drug-resistant* | U83.70^e^, U83.700^e^, U83.70+0^e^ |
| Multiple drug resistance | U83.71^e^, U83.710^e^, U83.71+0^e^ |
| Other and unspecified | U83.8, U83.80^e^, U83.8+0^e^, U83.9, U83.90^e^, U83.9+0^e^ |
| Unknown^f^ | Not possible to associate the resistance code to an organism |

^a^ Infections associated with medical device included infections on foreign body and infections associated with diagnostic procedures.

^b^ These codes are not specific to infection; they are considered as an infection if there is a micro-organism coded during the stay.

^c^ Bacteremia and sepsis

^d^ Codes specific to bacterial carriage were excluded

^e^ These codes are specific to the French version of ICD-10.

^f^ Stays with several resistance or with remaining non-concordant microorganism-resistance pairs were classified as “unknown”

Supplementary Table S2: Description of accepted bacteria-resistance couple.

|  | *Streptococci* | | | *Staphylococci* | | *Enterobacteriaceae* | Gram negative bacteria |
| --- | --- | --- | --- | --- | --- | --- | --- |
|  | *Streptococcus pneumoniae* | *Enterococcus*  (D group) | Others | *Staphylococcus aureus* | Others |  |  |
| Penicillin | X |  |  |  |  |  |  |
| Methicillin |  |  |  | X | X |  |  |
| Extended Spectrum β-Lactamase-producing |  |  |  |  |  | X | X |
| Other β-lactam resistance mechanism | X |  |  |  |  | X | X |
| Vancomycin and related |  | X |  |  |  |  |  |
| Quinolone | X |  | X | X | X | X | X |
| Multiple antibiotics | X | X | X | X | X | X | X |
| Emerging highly *drug-resistant* |  |  |  |  |  | X |  |
| Multiple drug resistance^a^ | X | X |  | X | X | X | X |
| Other and unspecified | X | X | X | X | X | X | X |

^a^ It was assumed that multidrug resistant bacteria refers to a bacterium resistant to at least 3 classes of antibiotic to which it is normally sensitive.

Supplementary Table S3: Description of the two groups *M+* and *M-*

|  | M+ | | M- | |
| --- | --- | --- | --- | --- |
| Patient characteristics | n | % | n | % |
| Sex |  |  |  |  |
| *Male* | 142247 | 40.5 | 387415 | 49.4 |
| Age (years) |  |  |  |  |
| *mean / SD* | 60.0 | 27.8 | 57.4 | 28.3 |
| Stay characteristics |  |  |  |  |
| Length of stay |  |  |  |  |
| *mean / SD* | 10.7 | 11.6 | 7.3 | 8.5 |
| Inhospital Mortality | 15877 | 4.5 | 41952 | 5.3 |
| Infection characteristics |  | |  | |
| Infection site |  |  |  |  |
| Urinary and genital tract | 179067 | 51.0 | 81771 | 10.4 |
| Lower respiratory tract | 30334 | 8.7 | 264616 | 33.7 |
| Gastrointestinal and abdominal | 17245 | 4.9 | 171339 | 21.9 |
| Skin and soft tissues | 18176 | 5.2 | 88945 | 11.3 |
| Primary blood infection^c^ | 20198 | 5.8 | 55546 | 7.1 |
| Material infection | 33136 | 9.4 | 18862 | 2.4 |
| Ear, nose and throat | 4248 | 1.2 | 43625 | 5.6 |
| Heart and mediastinum | 2773 | 0.8 | 20300 | 2.6 |
| Bone and joint | 5418 | 1.5 | 6843 | 0.9 |
| Infection during pregnancy | 17245 | 7.9 | 17106 | 2.2 |
| Infection in newborn | 11195 | 3.2 | 8900 | 1.1 |
| Eye | 750 | 0.2 | 3833 | 0.5 |
| Nervous system | 858 | 0.2 | 2336 | 0.3 |
